# An insulin-sensitive *Drosophila* insulin-like receptor mutant remodels methionine metabolism to extend lifespan

**DOI:** 10.1371/journal.pgen.1011640

**Published:** 2025-06-16

**Authors:** Marc Tatar, Wenjing Zheng, Shweta Yadav, Rochele Yamamoto, Noelle Curtis-Joseph, Shengxi Li, Lin Wang, Andrey A. Parkhitko

**Affiliations:** 1 Department of Ecology, Evolution and Organismal Biology, The Center for the Biology of Aging, Brown University, Providence, Rhode Island, United States of America; 2 Aging Institute of UPMC and the University of Pittsburgh, Pittsburgh, Pennsylvania, United States of America; 3 State Key Laboratory of Common Mechanism Research for Major Disease, Institute of Basic Medical Sciences, Chinese Academy of Medical Sciences and Peking Union Medical College, Beijing, China; Johns Hopkins University School of Medicine, UNITED STATES OF AMERICA

## Abstract

Insulin/insulin growth factor signaling is a conserved pathway that regulates lifespan across many species. Multiple mechanisms are proposed for how this altered signaling slows aging. To elaborate these causes, we recently developed a series of *Drosophila* insulin-like receptor (*dInr*) mutants with single amino acid substitutions that extend lifespan but differentially affect insulin sensitivity, growth and reproduction. Transheterozygotes of canonical *dInr* mutants (Type I) extend longevity and are insulin-resistant, small and weakly fecund. In contrast, a dominant mutation (*dInr*^353^, Type II) within the Kinase Insert Domain (KID) robustly extends longevity but is insulin-sensitive, full-sized, and highly fecund. We applied transcriptome and metabolome analyses to explore how *dInr*^353^ slows aging without insulin resistance. Type I and II mutants overlap in many pathways but also produce distinct transcriptomic profiles that include differences in innate immune and reproductive functions. In metabolomic analyses, the KID mutant *dInr*^353^ reprograms methionine metabolism in a way that phenocopies dietary methionine restriction, in contrast to canonical mutants which are characterized by upregulation of the transsulfuration pathway. Because abrogation of S-adenosylhomocysteine hydrolase blocks the longevity benefit conferred by *dInr*^353^, we conclude the methionine cycle reprogramming of Type II is sufficient to slow aging. Metabolomic analysis further revealed the Type II mutant is metabolically flexible: unlike aged wildtype, aged *dInr*^353^ adults can reroute methionine toward the transsulfuration pathway, while Type I mutant flies upregulate the transsulfuration pathway continuously from young age. Altered insulin/insulin growth factor signaling has the potential to slow aging without the complications of insulin resistance by modulating methionine cycle dynamics.

## Author summary

Mutations in the invertebrate insulin/IGF signaling system robustly extend lifespan. Yet these interventions often cause insulin resistance, reduce growth, and impair fertility. In contrast, a dominant gain-of-function mutation in the kinase insert domain of the Drosophila insulin/IGF receptor extends lifespan while maintaining insulin sensitivity, growth, or reproduction. Here, we demonstrate this unique mutation reprograms the methionine metabolism pathway in a way that mirrors dietary methionine restriction, which is known to extend lifespan in several animals including Drosophila and mice. Genetic epistasis analysis verifies this methionine cycle inhibition is an underlying cause for how the kinase insert domain mutation slows Drosophila aging while maintaining insulin-sensitivity, growth, and fecundity.

## Introduction

Mutations of the insulin-like receptor extend lifespan in *C. elegans* and *D. melanogaster*. Slow aging in these and related manipulations of the insulin/IGF pathway is proposed to arise through mechanisms that involve proteostasis, DNA repair, translation rate, innate immunity, reproduction, growth, and mitochondrial capacity [1–3]. This multiplicity of potential causes makes it challenging to evaluate which are required to slow aging from those that merely co-occur with altered insulin/IGF signaling or are secondary consequences of the slow aging. One case of such pleiotropy involves the *Drosophila* insulin-like receptor (*dInr*) where a series of single amino acid substitutions extends lifespan but differentially affect growth and reproduction [4]. ‘Type I’ mutations produce small, long-lived adults with low fertility, and where insulin weakly stimulates the phosphorylation of Akt. In contrast, a substitution in the kinase insert domain (KID) (‘Type II mutation’) robustly increases lifespan but produces full-sized adults with high fecundity, and where pAkt is strongly induced by insulin [4]. This mutation of the kinase insert domain provides an opportunity to dissect how the insulin-like receptor of *Drosophila* can slow aging without the infertility, impaired growth, and insulin resistance of canonical longevity mutations.

*Drosophila* has a single insulin-like receptor (*dInr*), as does *C. elegans* (*daf-2*). There are multiple insulin/IGF-like ligands for each of these receptors, encoded by seven loci in *Drosophila* [5] and by 40 loci in *C. elegans* [6,7]. Binding and activation studies confirm that *Drosophila* DILP2 and DILP5 are dInr agonists. Both stimulate the phosphorylation of Akt in a cell culture assay although along different time courses [8]. Structural analysis demonstrates three DILP5 ligands interact with dInr to produce an asymmetric *Ƭ*-shaped conformation, while a single DILP2 bound to dInr is predicted to induce an asymmetric conformation of the receptor [9]. Seven insulin-like ligands in *C. elegans* are categorized as strong agonists based on how they affect dauer formation, cell division, and metabolism; while 10 insulin-like peptides are seen as antagonists of Daf-2 function [6]. Given the pleiotropy of the *C. elegans* system, Gems and colleagues described how a range of *daf-2* mutations affected dauer, fertility, growth, and aging [10]. Alleles were categorized as Class 1 and Class 2 based on epistatic interactions with genes in the dauer formation pathway and by where their mutations fell within the Daf-2 receptor. Patel et al. [11] subsequently compared transcript profiles of *daf-2* alleles when combined with a mutation of the FOXO transcription factor *daf-16*. Among alleles that robustly extended lifespan, mutants varied in how they activated Daf-16 and by which genes they induced. Patel concluded that the site of amino acid substitution within the insulin-like receptor dictates phenotypes by altering the balance of the receptor intracellular signaling [11].

Yamamoto [4] conducted an allele series analysis of the *Drosophila* insulin-like receptor by evaluating how single amino acid substitutions variously affected lifespan, growth, and reproduction. Complementation analysis was used to screen a collection of alleles [12], and longevity candidates were subsequently regenerated by homologous recombination in a coisogenic background. Four alleles robustly extended male and female lifespan when combined as trans-heterozygotes. No alleles were viable as homozygotes, while one allele, *dInr*^353^, extended lifespan as a wildtype heterozygote (*dInr*^353^/*dInr*^wt^). All trans-heterozygotes produced small adults with reduced fecundity. In contrast, the growth and egg production of *dInr*^353^/*dInr*^wt^ adults were 1.6 greater than wildtype. Yamamoto evaluated the ability of these *dInr* genotypes to induce pAkt. Trans-heterozygotes weakly induced pAkt in an *ex vivo* insulin stimulation assay but *dInr*^353^/*dInr*^wt^ was as effective as wildtype [4].

The *dInr* longevity alleles differ by single amino acid substitutions. Alleles that extend lifespan only as trans-heterozygotes include a substitution in the extracellular Fibronectin III domain (*dInr*^E19^), a substitution in the intracellular tyrosine autoactivation loop (*dInr*^74^) and a substitution in the kinase domain C-terminal lobe (*dInr*^211^). We categorize these *Drosophila* insulin-resistant, growth-retarding, recessive longevity alleles as ‘Type I’. In contrast, the allele *dInr*^353^ is a substitution (Arg1466Cys) in the Kinase Insert Domain (KID), an unstructured peptide sequence of 27 residues that links the N- and C-lobes of the intracellular kinase domain. We categorize this *Drosophila* insulin-sensitive, growth-promoting longevity allele as ‘Type II’. Kinase insert domains occur in all tyrosine kinase receptors, while they vary in length and function [13]. Unlike typical tyrosine kinase receptors, the KIDs of insulin-like receptors are short, and their functions are largely unknown. *dInr*^353^ in *Drosophila* represents the first case of a non-pathogenic lesion in a KID of insulin-like receptors: it is a dominant allele that extends lifespan without insulin resistance, reduced growth or impaired fecundity. Here, we describe how *dInr* Type I and Type II alleles differentially affect the adult transcriptome and metabolome to understand the potentially unique mechanism by which *dInr*^353^ slows aging without insulin resistance.

## Results

### Transcriptional profiles of Type I *dInr*^*E19*^/*dInr*^*74*^ and Type II *dInr*^353^/*dInr*^wt^

**Fig 1:**
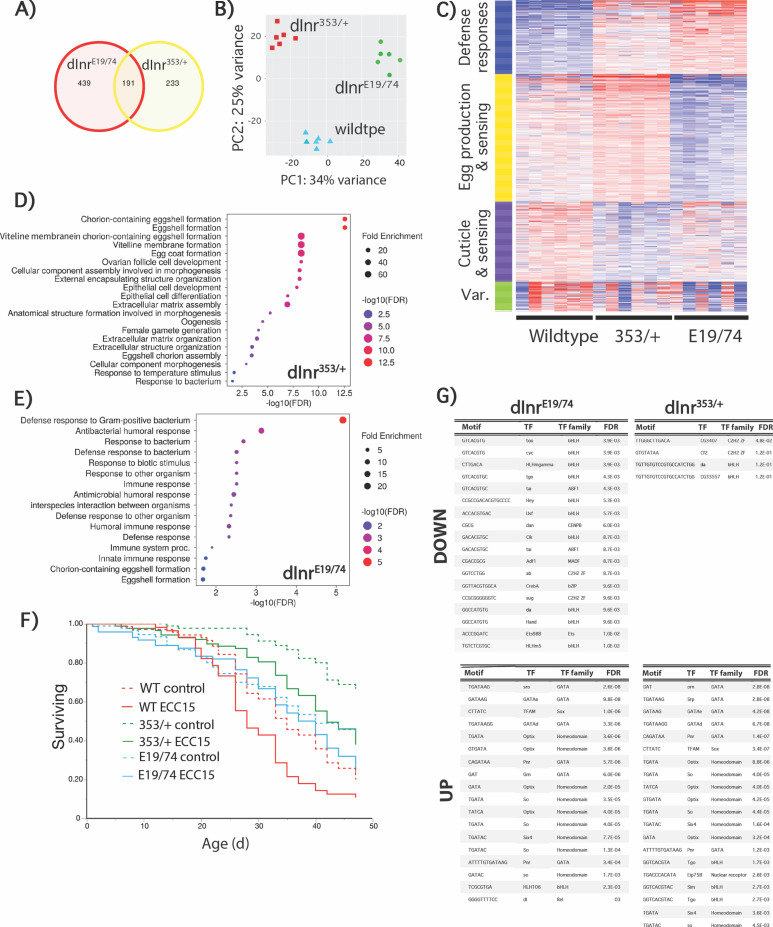
Transcriptional profiles of *dInr*^*E19*^/*dInr*^*74*^ and *dInr*^353^/*dInr*^wt^. **(A)** Venn diagram of transcripts altered in Type I *dInr*^*E19*^*/dInr*^*74*^ and Type II *dInr*^353^/*dInr*^wt^ genotypes. **(B)** Principal component analysis of transcripts profiled in *dInr*^*E19*^*/dInr*^*74*^ and *dInr*^353^/*dInr*^wt^. **(C)** Heatmap of transcripts altered in *dInr*^*E19*^*/dInr*^*74*^ and *dInr*^353^/*dInr*^wt^. **(D)** Gene set enrichment analysis of transcripts altered in *dInr*^353^/*dInr*^wt^. **(E)** Gene set enrichment analysis of transcripts altered in *dInr*^*E19*^*/dInr*^*74*^. **(F)** Survival of *dInr*^E19^/*dInr*^74^, *dInr*^353^/*dInr*^wt^, and coisogenic wildtype (*dInr*^wt/wt^) infected with *ECC15* bacteria. **(G)** Down- and up-regulated transcription factor binding sites in *dInr*^*E19*^*/dInr*^*74*^ and *dInr*^353^/*dInr*^wt^.

We conducted RNA-seq from somatic tissue of 10-day-old mated females of *dInr*^E19^/*dInr*^74^ (Type I), *dInr*^353^/*dInr*^wt^ (Type II), and coisogenic wildtype (*dInr*^wt/wt^ line ‘29B’) (Fig 1A and S1 Table). 862 transcripts were differentially expressed among the three genotypes (iDEP http://bioinformatics.sdstate.edu/idep96/). 191 differentially expressed genes relative to wildtype were common to *dInr*^E19^/*dInr*^74^ and *dInr*^353^/*dInr*^wt^. Relative to wildtype, adult *dInr*^E19^/*dInr*^74^ uniquely altered 439 genes, while 233 were uniquely expressed by *dInr*^353^/*dInr*^wt^. The first principal component axis separated all the genotypes, while on principal component axis 2, the two longevity genotypes were similar but distinct from wildtype (Fig 1B).

K-means clustering and gene set enrichment describe how these genotypes differ based on function (Fig 1C–1E and S2 Table). Type I *dInr*^E19^/*dInr*^74^ elevates defense response genes and reduces transcripts for egg production, consistent with their low fecundity [4]. In contrast, defense response is weakly amplified in *dInr*^353^/*dInr*^wt^, while this genotype increases transcripts associated with somatic activity to support egg production, consistent with their high fecundity. To evaluate the impact of altered defense gene expression, we orally infected adults with *ECC15* bacteria. Type I *dInr*^E19^/*dInr*^74^ adults resisted infection and survived as well as uninfected controls. In contrast, *dInr*^353^/*dInr*^wt^ suffered infection-induced mortality to the same extent as infected wildtype adults (Fig 1F). Thus, altered innate immunity is not necessary for *dInr* mutations to extend longevity.

Differences in mRNA among the genotypes arise in part from how *dInr* regulates transcription factors. We therefore used iDEP to identify transcription factor binding sites (Fig 1G) that are enriched in the mutants relative to wildtype. Four TF motifs were under-represented (down) in *dInr*^353^/*dInr*^wt^ including *daughterless* (*da*), which was the only site also under-represented in *dInr*^E19^/*dInr*^74^. The *dInr* mutants shared many over-represented (up) TF binding motifs relative to wildtype exception for *tango*, which was under-represented in *dInr*^E19^/*dInr*^74^ yet over-represented in *dInr*^353^/*dInr*^wt^, and four sites that were unique to either *dInr*^E19^/*dInr*^74^ (*HLH106*, *dl*) or *dInr*^353^/*dInr*^wt^ (*Eip75B*, *sim*). Notable for their absence were binding sites for the transcription factors FOXO and Aop (Anterior open), which are recognized to function downstream of insulin signaling in *Drosophila* to control aging [14,15]. Genetic epistasis analyses will be required to robustly evaluate if these expected transcription factors are required for the Type I or Type II *dInr* genotypes to extend lifespan.

Both *dInr* types altered the mRNA of genes previously associated with extended *Drosophila* lifespan. Up-regulated longevity genes in both *dInr*^353^/*dInr*^wt^ and *dInr*^E19^/*dInr*^74^ include *tequilla*, *Sodh-1*, and *Fbp1* [16–18]. Shared down-regulated mRNA transcripts include *ImpL2*, which binds insulin ligands and extends lifespan when over-expressed [19–21], and *la costa* (*lcs*), which is suppressed by AOP when the activity of this transcription factor contributes to extended lifespan [14,22].

Adults of *dInr*^E19^/*dInr*^74^ depress *dawdle* (*daw*) and adipokinetic hormone (*Akh*). *Dawdle* is modulated by FOXO when reduced insulin signaling extends lifespan [15]. Adipokinetic hormone has glucagon-like functions that enhance lifespan by regulating water homeostasis and metabolism [8,15,23]. Glycine N-methyltransferase (*gnmt*) was induced in *dInr*^E19^/*dInr*^74^ but not in *dInr*^353^/*dInr*^wt^. GNMT uses S-adenosylmethionine as a cofactor to methylate glycine into sarcosine [24]. Notably, over-expression of *gnmt* extends lifespan of wildtype adults, and the gene is required for reduced insulin activity (via a transgene for a dominant negative *dInr*) to slow aging [25,26]. We likewise find *dInr*^E19^/*dInr*^74^ increases insulin-like peptides *ilp3* and *ilp5* mRNA, indicating that these adults are hyper-insulinemic.

Few transcripts with recognized impact on aging change exclusively in *dInr*^353^/*dInr*^wt^. Rather, many DEGs of *dInr*^353^/*dInr*^wt^ are associated with egg production; however the transcription factor daughterless (*da*) is reduced only in *dInr*^353^/*dInr*^wt^, although its transcription factor binding site is under-represented in both types of *dInr*. *dInr*^353^/*dInr*^wt^ also upregulates several Tot secreted stress-response proteins (TotA, TotC, and TotX), while it downregulates the transcripts for the innate immune proteins DptA, Drosomycin, and Edin.

Overall, while Type I and Type II *dInR* mutants have several common downstream transcriptional impacts, the *dInr*^353^/*dInr*^wt^ mutant drives a program distinct from insulin-resistant Type I adults.

### Metabolomic profiles of *dInr*^*E19*^/*dInr*^*74*^ and *dInr*^353^/*dInr*^wt^

**Fig 2:**
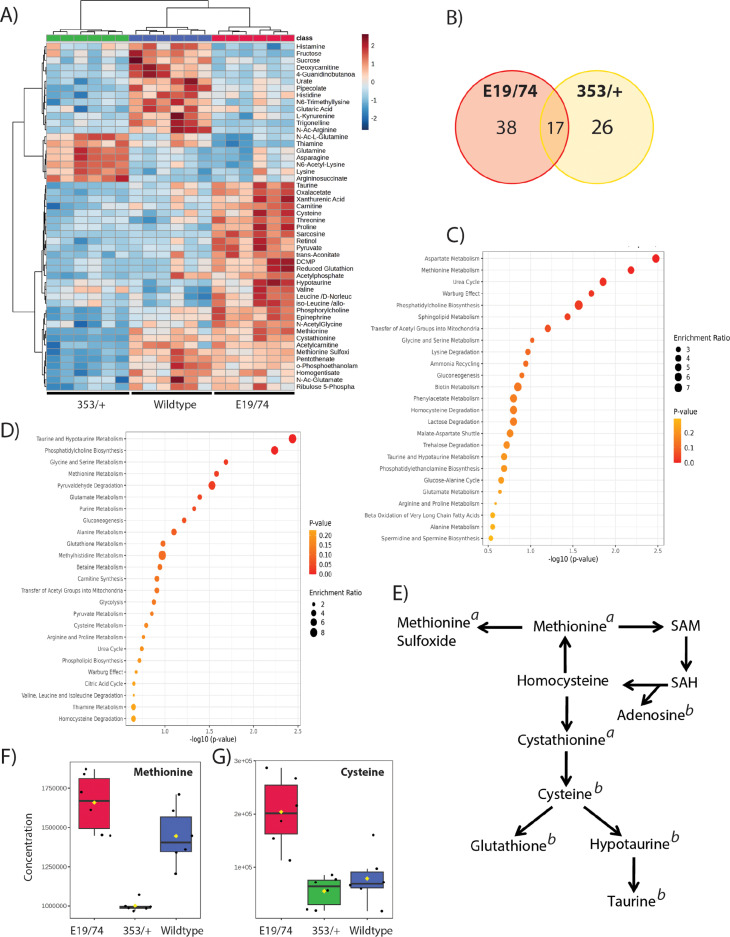
Steady state metabolomic profiles of 15-day old females of *dInr*^*E19*^/*dInr*^*74*^ and *dInr*^353^/*dInr*^wt^. **(A)** Heatmap of the most altered metabolites in *dInr*^*E19*^*/dInr*^*74*^ and *dInr*^353^/*dInr*^wt^. **(B)** Venn diagram of metabolites significantly altered in *dInr*^*E19*^*/dInr*^*74*^ and *dInr*^353^/*dInr*^wt^. **(C)** Metabolite set enrichment of metabolites altered in *dInr*^353^/*dInr*^wt^. **(D)** Metabolite set enrichment of metabolites altered in *dInr*^*E19*^*/dInr*^*74*^. **(E)** Scheme of methionine metabolism. Significantly (FDR < 0.1) altered metabolites relative to wildtype are marked with “*a*” for *dInr*^353^/*dInr*^wt^ and “*b*” for *dInr*^*E19*^*/dInr*^*74*^. **(F)** Relative levels of methionine in *dInr*^E19^/*dInr*^74^, *dInr*^353^/*dInr*^wt^, and coisogenic wildtype (*dInr*^wt/wt^). **(G)** Relative levels of cysteine in *dInr*^E19^/*dInr*^74^, *dInr*^353^/*dInr*^wt^, and coisogenic wildtype (*dInr*^wt/wt^).

To study the metabolic consequences of altered insulin receptor function, we performed targeted steady-state metabolite profiling of the insulin-resistant *dInr*^E19^/*dInr*^74^ and the insulin-sensitive *dInr*^353^/*dInr*^wt^ at different ages. Somatic tissues of 15-day-old and 30-day-old-females were analyzed, with six biological replicates (each of 10 females without ovaries) of both genotypes. We first considered the 15-day-olds alone. Here, the three genotypes can be distinguished with principal component analysis (PCA) (S1 Fig) based on the 170 detected metabolites within the target set (361 metabolites). Relative to wildtype, 26 metabolites were uniquely altered in *dInr*^353^/*dInr*^wt^ and 38 were differentially abundant just in *dInr*^E19^/*dInr*^74^ (FDR < 0.05). Seventeen metabolites were similarly affected in the same direction in both genotypes (Fig 2A and 2B and S3 Table).

Metabolite Set Enrichment Analysis distinguishes pathways affected by each mutant. Type II *dInr*^353^/*dInr*^wt^ impacts ‘aspartate metabolism’ and ‘methionine metabolism’. Insulin-resistant *dInr*^E19^/*dInr*^74^ mutants affect ‘taurine and hypotaurine metabolism’, and ‘phosphatidylcholine biosynthesis’ (Fig 2C and 2D). Notably, these longevity genotypes intersect at methionine metabolism (Fig 2E) but in strikingly different ways. At 15 days of age, *dInr*^353^/*dInr*^wt^ has reduced steady-state methionine, methionine sulfoxide, and cystathionine (Figs 2E, 2F and S1), while *dInr*^E19^/*dInr*^74^ has elevated cysteine, hypotaurine, taurine, and adenosine, and reduced glutathione (Figs 2E, 2G and S1). Sarcosine is also strongly enriched (7-fold) in *dInr*^E19^/*dInr*^74^ yet is less abundant in *dInr*^353^/*dInr*^wt^ adults. Sarcosine is notable because this metabolite is generated by GNMT, and we found *gnmt* mRNA is markedly greater in *dInr*^E19^/*dInr*^74^ (S1 Table). Previous work has established that *gnmt* plays a role in how repressed insulin signaling extends lifespan [25,26]. Overall, Type I and Type II *dInr* mutants produce different patterns of reprogrammed methionine metabolism.

### Age dynamics of *dInr*^*E19*^/*dInr*^*74*^ and *dInr*^353^/*dInr*^wt^ metabolic profiles

**Fig 3:**
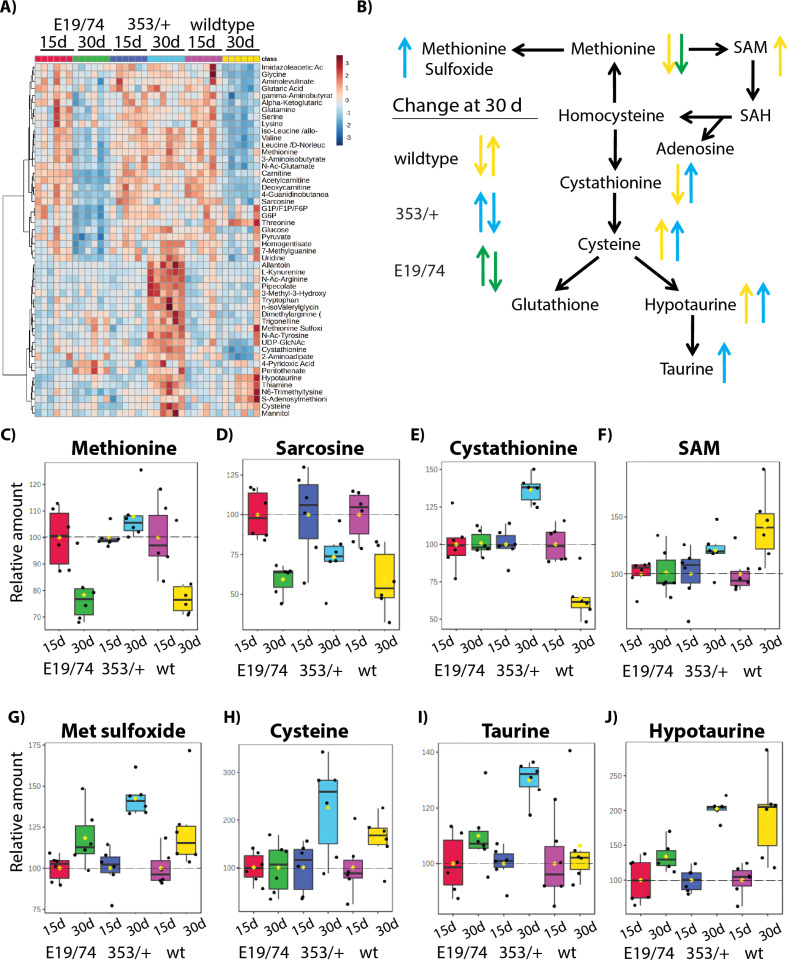
Age-associated metabolic profiles of *dInr*^*E19*^/*dInr*^*74*^ and *dInr*^353^/*dInr*^wt^. **(A)** Heatmap of age-associated metabolites among wildtype (*dInr*^wt/wt^), *dInr*^*E19*^*/dInr*^*74*^, or *dInr*^353^/*dInr*^wt^. **(B)** Methionine metabolism network where significant age-associated changes at day 30 are indicated for each genotype. **(C-J)** The level of methionine metabolism at day 30 relative to the level within each genotype at day 15 (all values normalized to the mean at day 15): (**C**) methionine, (**D**) sarcosine, (**E**) cystathionine, **(F)** SAM, (**G**) methionine sulfoxide, (**H**) cysteine, (**I**) taurine, and (**J**) hypotaurine.

Metabolic profiles change with age [27], including the profile of methionine metabolism in *Drosophila* [28,29]. We therefore analyzed how aging affects metabolic changes in *dInr*^wt^/*dInr*^wt^, *dInr*^*E19*^*/dInr*^*74*^*, and dInr*^353^/*dInr*^wt^ females. Fig 3 (Fig 3C–3J) normalizes metabolites to their level at 15-days old; day 15 is set at 100%, and the value at day 30 reflects the change relative to day 15. The direction of these changes within the methionine network is summarized in Fig 3B. Relative to young adults, methionine, sarcosine, and cystathionine decreased with age in wildtype *dInr*^wt^/*dInr*^wt^ (Fig 3C, 3D and 3E), as previously reported for wildtype *OregonR*, *yw*, and *w*^*1118*^ flies [28,30]. The elevated methionine and sarcosine seen in young *dInr*^E19^/*dInr*^74^ adults decreased with age (Fig 3C and 3D). In contrast, the steady-state level of methionine was low in young *dInr*^353^/*dInr*^wt^ adults and remained low in aged adults (Fig 3C). SAM increased with age in wildtype flies but remained constant across age in *dInr*^E19^/*dInr*^74^ and *dInr*^353^/*dInr*^wt^ (Fig 3F). The level of methionine sulfoxide was relatively low in young *dInr*^353^/*dInr*^wt^ adults and significantly increased as this genotype aged, but the metabolite remained constant in *dInr*^E19^/*dInr*^74^ and wildtype adults as they aged (Fig 3G).

There are notable differences among genotypes in how metabolites of the transsulfuration pathway change with age. Aged wildtype flies have reduced levels of cystathionine but elevated hypotaurine (Fig 3E and 3H–3J), while these metabolites were initially elevated in young *dInr*^E19^/*dInr*^74^ adults and remained high in aged adults (Fig 3E and 3H–3J). In contrast, all assayed transsulfuration metabolites were initially low but then markedly increased with age in *dInr*^353^/*dInr*^wt^ adults (Fig 3E and 3H–3J).

The *dInr* genotypes have different age-associated changes in methionine metabolism. Female *dInr*^E19^/*dInr*^74^ mutants enhance the transsulfuration pathway as young adults and maintain this level with age. Young insulin-sensitive *dInr*^353^/*dInr*^wt^ adults produce few transsulfuration metabolites but increase these molecules as they age.

### Methionine cycle dynamics of *dInr*^*E19*^/*dInr*^*74*^ and *dInr*^353^/*dInr*^wt^

Tracking ^13^C can estimate the transition rates across steps in the methionine cycle and quantify the relative flow of methionine derivatives toward nucleotides, polyamines, and the transsulfuration pathway [31,32]. In this approach, carbon isotope-labeled nutrients are fed to adults, and the source of carbons within specific metabolites is estimated from the quantity of labeled and unlabeled carbons [33–35].

**Fig 4:**
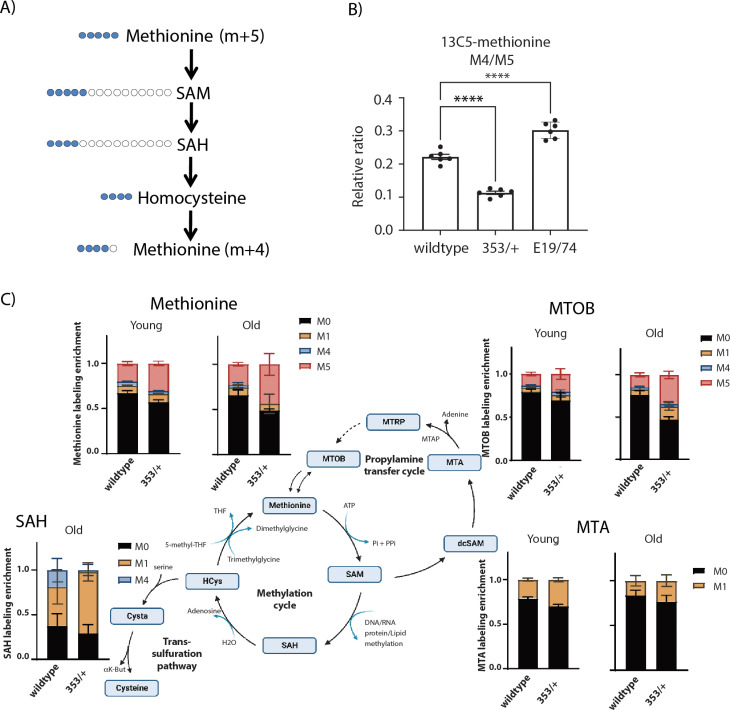
Methionine dynamics from isotope tracking in *dInr*^*E19*^/*dInr*^*74*^ and *dInr*^353^/*dInr*^wt^. **(A)** Scheme of methionine labeling. Blue marks the labeled carbons in each metabolite derived from the labeled dietary methionine, which has five ^13^C (m + 5). **(B)** The ratio between methionine (m + 4) recovered from the methionine cycle and methionine (m + 5) derived from the diet. Differences among the ratios reflect significant differences (ANOVA test with posthoc correction for multiple hypothesis testing) in the activity of the central methionine cycle among wildtype (*dInr*^wt/wt^), *dInr*^*E19*^*/dInr*^*74*^, and *dInr*^353^/*dInr*^wt^. ***, p < 0.001; ****, p < 0.0001. **(C)** The distribution of labeled methionine in young and old female wildtype (*dInr*^wt/wt^), *dInr*^*E19*^*/dInr*^*74*^, and *dInr*^353^/*dInr*^wt^, referenced to the methionine cycle, polyamine cycle and transsulfuration pathway. The number of labeled carbons in each metabolite is indicated as M0-M5. Bars reflect the relative proportion of each labeled isoform (labeling enrichment) within metabolites at each age and genotype.

We fed young *dInr* adults with stable isotope-labeled methionine (^13^C5-methionine, five labeled carbons, m + 5) and measured how these carbons were incorporated into methionine that is regenerated from homocysteine, which is seen as methionine m + 4 (^13^C4-methionine, four labeled carbons: m + 4) [28,30] (Fig 4A). We find that the ratio of methionine m + 4/m + 5 is higher in *dInr*^E19^/*dInr*^74^ than in wildtype (Fig 4B), indicating that the activity of the central methionine cycle is greater in these insulin-resistant females than in wildtype. This high activity can account for the elevated steady-state sarcosine and transsulfuration pathway products of the Type I adults and implies that somatic cells of these mutants may need to cope with abundant methionine. In contrast, the ratio of m + 4/m + 5 methionine is reduced in *dInr*^353^/*dInr*^wt^ relative to wildtype (Fig 4B), indicating the somatic tissue of these adults has lower activity through their central methionine cycle. Consequently, they produce less Hcy and recycle less methionine.

Although we cannot directly estimate flux to the transsulfuration pathway, it seems unlikely that methionine in *dInr*^353^/*dInr*^wt^ is routed through that pathway because we do not observe elevated taurine or glutathione in the steady-state metabolome. Likewise, roles for the salvage pathway are also difficult to evaluate because metabolites were not well represented in our steady-state data. Some insight is gained, however, from methionine carbons traced into the salvage pathway of young and aged flies. Dietary methionine is first converted to S-adenosyl-L-methionine (SAM), which can be decarboxylated by AdoMet decarboxylase into decarboxylated SAM (dcSAM), an aminopropyl group donor. Spermidine synthase and spermine synthase use this aminopropyl donor to convert putrescine to spermidine and spermine, with dcSAM converted to MTA. MTA then undergoes six enzymatic steps to regenerate methionine, with 4-methylthio-2-oxobutanoic acid (MTOB) as one intermediary. In *dInr*^353^/*dInr*^wt^ adults, we see that MTA and MTOB are labeled with one carbon (m + 1) derived from methionine, which can arise from m + 4 or m + 5 labeled methionine (Fig 4C). Furthermore, we see a significantly elevated fraction of m + 1 labeled MTA (in young and old flies) and of m + 1 labeled MTOB (in old flies), suggesting the *dInr*^353^/*dInr*^wt^ genotype may indeed route methionine towards polyamine production.

Our technical platform did not detect differential labeling of SAM and cystathionine. Likewise, we could detect labeled SAH only in old flies, perhaps because SAH is intrinsically elevated with age [28]. SAH generated directly from m + 5 or m + 4 methionine is labeled as m + 4 SAH. SAH generated from methionine that passed through the salvage pathway is labeled as m + 1 SAH (via m + 1 methionine). Notably, we found an elevated fraction of m + 1 SAH in *dInr*^353^/*dInr*^wt^ adults and almost no fraction of m + 4 SAH. These results imply that carbons from methionine in *dInr*^353^/*dInr*^wt^ adults move through the salvage pathway first and then enter the methionine cycle, consistent with the m + 1 labeling of salvage pathway intermediaries (MTA and MTOB).

Overall, *dInr*^353^/*dInr*^wt^ adults have two unique features of methionine metabolism: their central methionine cycle has reduced activity, as occurs in dietary methionine restriction, and they activate the methionine salvage pathway, which is expected to generate polyamines.

### Interactions between *dInr*^353^/*dInr*^wt^ and genes of the methionine cycle

**Fig 5:**
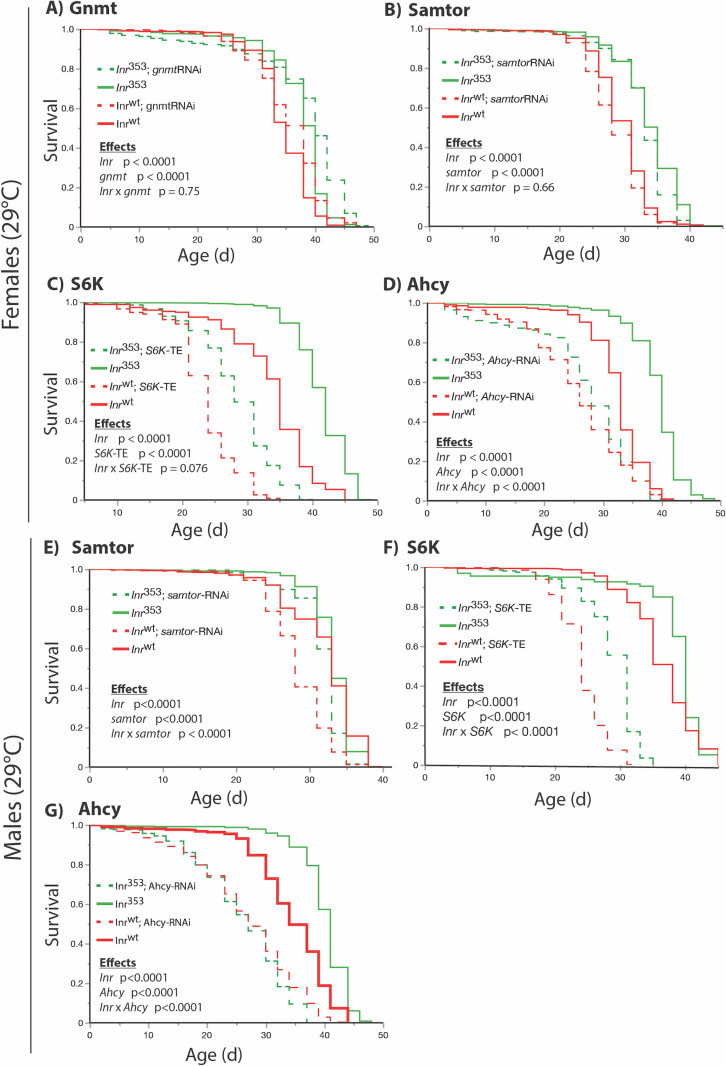
Epistasis analysis for interactions between elements of the methionine cycle and the *dInr* KID mutant that affect survival. Kaplan-Meier plots and Cox proportional hazard to evaluate the epistatic impact on survival of the *dInr* genotype, the targeted methionine cycle-associated gene and the interaction between these factors. **(A)** Survival of female wildtype (*dInr*^wt/wt^) and *dInr*^353^/*dInr*^wt^ combined with *gnmt* RNAi. **(B)** Survival of female wildtype (*dInr*^wt/wt^) and *dInr*^353^/*dInr*^wt^ combined with *samtor* RNAi. **(C)** Survival of female wildtype (*dInr*^wt/wt^) and *dInr*^353^/*dInr*^wt^ combined with *S6K-*activated transgene. **(D)** Survival of female wildtype (*dInr*^wt/wt^) and *dInr*^353^/*dInr*^wt^ combined with *Ahcy13* RNAi. **(E)** Survival of male wildtype (*dInr*^wt/wt^) and *dInr*^353^/*dInr*^wt^ combined *samtor* RNAi. **(F)** Survival of male wildtype (*dInr*^wt/wt^) and *dInr*^353^/*dInr*^wt^ combined with *S6K-*activated transgene. **(G)** Survival of male wildtype (*dInr*^wt/wt^) and *dInr*^353^/*dInr*^wt^ combined with *Ahcy13* RNAi.

Previous work established that reduced insulin signaling confers longevity in part because it induces *gnmt*, which itself is required for *dInr* genotypes to extend lifespan [25,26]. Consistent with those reports, we find that *dInr*^E19^/*dInr*^74^ increases *gnmt* mRNA and sarcosine, the product of GNMT enzymatic activity. In contrast, these features were not seen in *dInr*^353^/*dInr*^wt^, and correspondingly, knockdown of *gnmt* mRNA in *dInr*^353^/*dInr*^wt^ did not inhibit the exceptional longevity of the Type II mutant (Fig 5A). We therefore tested other components of the methionine cycle. As noted, S-adenosyl-methionine (SAM) was reduced in *dInr*^353^/*dInr*^wt^. SAM has been recently shown in mammalian cells to modulate TOR activity by disrupting the SAMTOR-GATOR1 complex [36], although a more recent report suggests a gene called ‘*unmet*’, not SAMTOR, is the SAM sensor in *Drosophila* [37]. In mammals, reduced SAM promotes the association of SAMTOR with GATOR1 and inhibits TORC1. Thus, reduced SAM may promote longevity by activating SAMTOR. Based on the initial report of SAMTOR, we investigated the interaction of *samtor* and *dInr*^353^/*wt* in *Drosophila* by depleting *samtor* in *dInr* mutant and wildtype adults. This did not diminish the extended longevity of *dInr*^353^/*dInr*^wt^ females (Fig 5B) while in males, *samtor*-RNAi alone repressed survival in wildtype flies (Fig 5E). To test if *dInr*^353^ otherwise interacts with TOR, we overexpressed activated S6K (UAS-S6K-TE) in *dInr*^353^ and wildtype adults. This manipulation depressed survival in all genotypes but did not eliminate the relative longevity benefit of *dInr*^353^ (Fig 5C and 5F). Downstream in the central methionine cycle, S-adenosyl-homocysteine (SAH) is generated when SAM contributes a methyl group to substrates [38]. SAH itself is an inhibitor of SAM-dependent methylation and is therefore robustly regulated by the activity of AHCY [39]. Here, we find that depletion of *Ahcy13* in the *dInr*^353^/*wt* background eliminates the survival advantage of the *dInr* mutant relative to similarly treated wildtype. While this depletion reduces the lifespan of all genotypes, as anticipated from previous work [28], we conclude that *dInr*^353^/*wt* requires *Ahcy13* to extend lifespan relative to *dInr* wildtype. Reduced flux through SAH is required for the *dInr*^353^/*dInr*^wt^ mutant to extend lifespan: Type II *dInr*^353^/*dInr*^wt^ slows aging by reprogramming the methionine cycle.

## Discussion

### Type I and II *dInr*

Type I and II insulin-like receptor mutants extend lifespan, yet these genotypes differ in many ways. Type I *dInr* genotypes are manipulations that reduce insulin signaling and extend lifespan but retard growth, slow development, repress fecundity, and cause insulin resistance. Genetically, they are homozygous lethal and extend longevity only as transheterozygotes. Yamamoto et al. [4] identified three alleles with these characteristics: *dInr*^E19^, *dInr*^211^, and *dInr*^74^. The *dInr*^E19^ allele was originally reported to increase lifespan and to reduce insulin receptor phosphorylation when combined with an enhancer trap insertion in the first coding exon [40]. The *dInr*^E19^ allele substitutes aspartic acid for valine in the linker sequence between the L2 and FnIII-1 ectodomains. *dInr*^74^ generates a missense mutation in the conserved activation loop of the intracellular kinase domain and likely reduces the ability of the kinase to be transactivated. The *dInr*^211^ allele substitutes glycine with arginine at residue 1598 of the kinase domain C-terminal lobe. It extends lifespan when combined with *dInr*^E19^ or with *dInr*^74^, where all transheterozygotes are insulin resistant.

In contrast, the Type II *dInr* mutant of the KID, when present as a heterozygote with a wildtype allele (*dInr*^353^/*dInr*^wt^), has normal growth, high fecundity, robust insulin sensitivity, and extended lifespan. KIDs are unstructured peptide sequences that link the kinase domain N- and C-lobes in tyrosine kinase receptors [13]. KIDs often contain binding motifs for adaptor proteins, although none are reported in the KID of Drosophila dInr, which is relatively small (27 residues), or in the KIDs of mammalian IR and IGF1R (both with 15 residues). These insulin/IGF KIDs have conserved residues at the start of the KID, including the arginine in *Drosophila* (R1466) that extends longevity in *dInr*^353^ when substituted for cysteine. No function to date has been identified for the KID of any insulin/IGF-like receptor. We therefore characterized transcriptional and metabolomic profiles of the Type II *dInr* relative to wildtype and the Type I mutants. We found the altered KID of dInr produces a methionine restriction-like state and showed that this mutant receptor genetically interacts with a key enzyme of the methionine cycle to slow aging.

### Transcriptional profiles of insulin-like receptor genotypes

*C. elegans* and *Drosophila* have been characterized for how mutant insulin receptors (*daf-2*, *dInr*) impact mRNA profiles and enrich for Daf-16/FOXO transcription factor binding sites [15,41]. Working with variants of *daf-2*, Patel et al. [11] described how different alleles impact mRNA levels. The weak allele *daf-2*(m577) modestly increases *C. elegans* lifespan and mildly affects the temperature at which animals enter dauer, while the often-studied *daf-2*(e1370) allele robustly increases lifespan, strongly affects dauer, and reduces feeding and movement [10]. Patel et al. [11] discriminated many differentially expressed genes among these longevity alleles and how these patterns depended on *daf-16*. Transcripts upregulated by *daf-2*(e1370) that were not affected in *daf-2*(m577) included small heat shock proteins, the insulin ligand *ins-22,* and the nuclear hormone receptor *nhr-206*. These genes may contribute to how *daf-2*(e1370) slows aging in ways that are distinct from the action of the *daf-2*(m577) allele.

Here, we discriminate among the differentially expressed genes of *Drosophila dInr* longevity mutants. Type I *dInr*^E19^/*dInr*^74^ and Type II *dInr*^353^/*dInr*^wt^ together expressed 862 different genes relative to wildtype. The Type II KID mutant *dInr*^353^/*dInr*^wt^ uniquely altered 233 transcripts that were not seen to change in the Type I mutants. Principal components axis 1 (PC1) partitioned all the *dInr* genotypes, while the mutant genotypes were similar on PC2 but together distinct from wildtype. Type I and Type II *dInr* mutants produce common and unique transcriptional changes that may contribute to extended lifespan.

*Tequila* is one transcript elevated in both *dInr* genotypes. Previous work found that a *tequila* hypomorph extended *Drosophila* lifespan, perhaps because it reduces neuronal insulin secretion [17]. In contrast, the *dInr* mutants may elevate *tequila* to increase insulin secretion as a mechanism to compensate for their mutated receptor function. Compensation may also explain why *ImpL2* is reduced in both genotypes: ImpL2 is a peptide-binding protein that inhibits the biological availability of *Drosophila* insulin-like peptides [19]; limiting this inhibitor may increase the bioavailability of insulin ligand. Consistent with these explanations, mRNA for insulin-like ligands *ilp3* and *ilp5* is elevated in Type I, although not in Type II. Overall, Type I *dInr* adults in particular have three features associated with human pre-diabetes: hyperinsulinemia, mechanisms to increase insulin peptide bioavailability, and impaired insulin receptor sensitivity [4].

Enzymes that mediate oxidative stress are elevated in many longevity-conferring manipulations of *Drosophila* and *C. elegans* [18], and we see elevated *Sod-1* in both types of long-lived *dInr* mutants. However, whether increased Sod-1 is sufficient to slow aging in *Drosophila* or mice is unresolved because its over-expression does not consistently extend lifespan [42]. Finally, fat body protein (*fbp*) was elevated in both types of *dInr* mutants. Fat body protein was recently implicated as a factor that limits lifespan when induced by juvenile hormone in young adults, while transiently blocking *fbp* in young adults improves life expectancy [16]. Thus, increased *fbp* in long-lived *dInr* genotypes, as seen here, is somewhat counter to expectations.

Few previously recognized aging-associated genes changed only in the *dInr*^353^/*dInr*^wt^ adults. One exception involves the transcription factor *daughterless* (*da*): *da* expression decreased, and we found few binding sites for this transcription factor among the differentially expressed genes of *dInr*^353^/*dInr*^wt^. Notably, depletion of the *daughterless* ortholog HLH-2 in *C. elegans* extends nematode lifespan [43].

There is a striking difference in innate immune-related genes among the *dInr* longevity genotypes. In previous studies, the expression of antimicrobial peptides and peptidoglycan recognition proteins declines with age, while dietary and genetic manipulations that extend *Drosophila* longevity often maintain these defenses [44]. These observations suggest that elevated innate immunity across age favors *Drosophila* longevity. Here, we see innate immune-associated genes are elevated in Type I *dInr*^E19^/*dInr*^74^ but not in Type II, and these expression differences are functional because they correspond with the ability of adults to resist oral infection with *ECC15* bacteria. This outcome demonstrates that elevated innate immunity is not necessary for altered insulin signaling to extend lifespan.

Overall, while both *dInr*^E19^/*dInr*^74^ and *dInr*^353^/*dInr*^wt^ extend lifespan, the genotypes express sets of distinct and similar genes. Remarkably, their differences are caused by the placement of single amino acid substitutions within the insulin-like receptor. Deeper analysis will be required to understand how these variants differentially transduce through signaling cascades, including the roles of the transcription factors FOXO and Aop.

### Methionine metabolism of insulin-like receptor mutants

#### Type I *dInr*^E19^/*dInr*^74^.

We detected 170 metabolites from *dInr Drosophila* mutants assessed from a panel of 360 compounds. Sarcosine showed the most prominent difference: relative to wildtype, sarcosine was sevenfold higher in Type I adult soma. Sarcosine is produced when glycine is methylated by GNMT using S-adenosyl-methionine (SAM) as a substrate, and we found transcripts of *gnmt* were correspondingly elevated in *dInr*^E19^/*dInr*^74^. Serum sarcosine is normally reduced during mammalian aging, while it is increased in rodents when dietary restriction extends lifespan [45]. In cells and rats, sarcosine can induce autophagy and repress the activity of TOR [45]. In *Drosophila*, *gnmt* is regulated by the insulin-responsive transcription factor FOXO, and lifespan is increased when *gnmt* is over-expressed, while *gnmt* is required for a dominant-negative *dInr* transgene to extend lifespan [26].

Elevated sarcosine in the soma of adult suggests that the cells of *dInr*^E19^/*dInr*^74^ are processing excess methionine, perhaps to keep SAM within homeostatic tolerance. SAM itself may impact aging in several ways. Aside from the conversion of glycine into sarcosine, SAM is the key substrate for methylation of lipids, proteins, and nucleotides, where such modifications could produce myriad impacts on aging [38]. SAM is also an entry molecule for the polyamine cycle when it is decarboxylated into an aminopropyl donor to produce spermidine and spermine. These polyamines are implicated in retarding aging by modulating autophagy and lysosome biogenesis [46–48]. Finally, SAM in mammalian cells represses SAMTOR, which otherwise represses the activity of TOR [36]. Thus, the supply of SAM modulates the activation of mTORC1 and this may thereby impact aging [36].

Donation of a methyl group by SAM produces SAH that is converted to homocysteine. Homocysteine is converted back to methionine or irreversibly directed into the transsulfuration pathway, where its products may promote longevity by reducing ROS-associated damage [49]. In *Drosophila*, longevity conferred by dietary restriction is restored to wildtype by inhibiting the rate-limiting transsulfuration pathway enzyme cystathionine β-synthase [50].

Aging itself alters the steady-state levels of methionine cycle metabolites, as previously seen in *Drosophila* and in mammalian serum [51]. We find that Type I and wildtype adults have reduced somatic methionine and sarcosine at 30 days relative to 15 days, yet they retain transsulfuration products at the levels expressed at a younger age. These dynamics suggest that while aging reduces the absolute levels of methionine and SAM, cells in the older Type I adults retain the ability to distribute carbons into the transsulfuration pathway.

We traced the fate of labeled carbons acquired from dietary methionine to verify that young Type I adults have elevated methionine cycle activity, as suggested by their high level of sarcosine. When dietary methionine (labeled as m + 5) traverses the central methionine cycle, it produces SAH with four labeled carbons that can be recycled to methionine, which is then labeled as m + 4. The ratio of m + 4 to m + 5 methionine therefore estimates the flow of carbons across the central methionine cycle [30]. Type I female *dInr*^E19^/*dInr*^74^ adults have strikingly greater m + 4/m + 5 ratios relative to wildtype, indicating they metabolize a larger portion of methionine into SAH, which is returned to methionine or directed into the TSP.

#### Type II (KID) *dInr*^353^/*dInr*^wt^.

Relative to Type I mutants, *dInr*^353^/*dInr*^wt^ affects the methionine cycle in remarkably different ways. The young adult soma has less methionine and sarcosine than wildtype, and transcripts for *gnmt* are not elevated. Young adults have less TSP products including methionine sulfoxide, cystathionine, cysteine, and taurine. Relative to wildtype, there is less SAM but the same amount of SAH. The impact of age (30 days) upon these metabolites is also distinct. Unlike wildtype and *dInr*^E19^/*dInr*^74^, *dInr*^353^/*dInr*^wt^ adults retain their young (low) level of methionine but increase the amount of transsulfuration pathway products, including cystathionine, methionine sulfoxide, cysteine, taurine, and hypotaurine.

We hypothesize these patterns arise from the high level of egg production seen in young *dInr*^353^/*dInr*^wt^ females. Young *dInr*^353^/*dInr*^wt^ females are 1.6 times more fecund than wildtype [4], and this activity might allocate dietary methionine into eggs rather than to somatic tissue. Old females produce few eggs, and dietary methionine may then be available for the TSP of somatic cells. In this model, the survival mechanisms supporting *dInr*^353^/*dInr*^wt^ longevity are biphasic: at a young age, it involves mechanisms produced by low somatic methionine, while at later ages, it involves activity of the TSP. The model contrasts with the results of Wei et al. [30], where dietary methionine restriction (MetR) elevated the m + 4/m + 5 ratio of methionine, but we find *dInr*^353^/*dInr*^wt^ decreases this ratio. This difference may arise because dietary methionine restriction represses egg production, which may be sufficient to elevate the metabolites of the TSP and corresponds to the pattern we see for *dInr*^E19^/*dInr*^74^. Type II *dInr*^353^/*dInr*^wt^, in contrast, is very fecund and this may reduce the ratio methionine m + 4/m + 5 ratio.

Isotope tracing of dietary methionine helps substantiate these interpretations. Young adult *dInr*^353^/*dInr*^wt^ flies have a low m + 4/m + 5 methionine ratio, along with low steady-state TSP. These trends indicate that there is relatively low activity of the methionine cycle beyond SAM, or that SAM is flowing into the polyamine pathway. Our data suggest that *dInr*^353^/*dInr*^wt^ directs SAM to the polyamine pathway because we find elevated m + 1 MTOB and m + 1 MTA in both young and old Type II adults. Although the proportion of m + 5 methionine increases in old (30d) *dInr*^353^/*dInr*^wt^ adults, we could not calculate m + 4/m + 5 because the level of m + 4 methionine is negligible. These data suggest that carbons from dietary methionine are shunted into the salvage pathway (indicated by increased m + 1 labeling in MTA, MTOB, and SAH) to become a resource for polyamines. Considering the reported pro-longevity effects of spermidine [47,48,52], our observations may partially explain how *dInr*^353^/*dInr*^wt^ is long-lived. Young *dInr*^353^/*dInr*^wt^ adults appear to experience restricted somatic methionine that impedes activity of the central methionine cycle while they increase the flow of methionine into the salvage pathway.

### Methionine restriction and aging

Dietary methionine restriction (MetR) slows aging across many species, including rodents and *Drosophila* [53–56]. Likewise, knockdown of *C. elegans* S-adenosyl methionine synthetase (*sams-1)* increases adult lifespan [57], and *Drosophila* lifespan is increased by 25% by expressing an exogenous Methioninase that degrades methionine [29]. MetR decreases levels of intermediates in the methionine metabolism pathway, including methionine, methionine sulfoxide, SAM, SAH, cystathionine, and 5’-methylthioadenosine (MTA) [29,30,58]. Profiles of methionine metabolism are also altered in long-lived species and strains, such as naked mole-rats [59], flies selected for delayed reproductive senescence [28,60], and Ames mice [61]. Across these cases, methionine restriction is thought to slow aging through many potential avenues, including protein and nucleotide methylation, polyamine production, protein translation, and energetic or oxidative status [51].

Steady-state metabolomics and tracing analysis demonstrate that young Type II adults have attributes of methionine restriction. The long-lived *dInr*^353^/*dInr*^wt^ adults have less methionine and less steady-state methionine sulfoxide. Type II adults also have increased m + 1 labeling in the salvage pathway and at SAH, and elevated transsulfuration metabolites as they age.

Genetic epistasis analysis confirms that *dInr*^353^/*dInr*^wt^ extends longevity because it modifies methionine metabolism. Unlike *gnmt*, which has been reported to be required for repressed insulin signaling to slow aging [25,26], the extended longevity of *dInr*^353^/*dInr*^wt^ is independent of *gnmt*. As well, *dInr*^353^/*dInr*^wt^ did not interact with SAMTOR or S6K to slow aging, suggesting its impact is independent of TOR (although the utility of testing SAMTOR in Drosophila is ambiguous). Rather, *dInr*^353^/*dInr*^wt^ requires *ahcy13*. AHCY-13 encodes SAHH (S-adenosylhomocysteine hydrolase), which converts SAH into homocysteine. Emerging work shows that AHCY itself modulates aging: *Drosophila* lifespan was increased by downregulating genetic repressors of AHCY (AhcyL1, AhcyL2) [28], and *C. elegans* lifespan was increased by a deficiency of AHCY-1 that increases the ratio of SAH to SAM [62]. Cells normally maintain low concentrations of SAH because it is an inhibitor of SAM-dependent methylation. Inhibition of AHCY blocks SAH clearance and prevents the flux of methionine through the methionine cycle and into the transsulfuration pathway. We have previously demonstrated that downregulation of Ahcy13 in *Drosophila* upregulates SAH levels [28], which strongly inhibits methionine flux. By inhibiting Ahcy13 in wildtype and *dInr*^353^ flies, we asked if the observed changes in methionine metabolism are required for the extended lifespan of *dInr*^353^/*dInr*^wt^. Depleting *ahcy13* shortens *dInr*^353^/*dInr*^wt^ lifespan to the level of similarly manipulated wildtype controls. We conclude that *dInr*^353^/*dInr*^wt^ extends longevity, in part, because it alters methionine metabolism. This metabolic programming has three impacts: i) methionine is routed towards the salvage/polyamine pathway; ii) the flux of the central via methionine cycle is reduced, and iii) old adults can upregulate products of the transsulfuration pathway.

### Alternative modes of longevity control by insulin/IGF signaling

Canonical Type I insulin mutants elevate the products of the TSP relative to wildtype at both young and older ages. These mutants have reduced fecundity, which perhaps directs methionine from eggs into the TSP of somatic cells. Thus, the *dInr*^E19^/*dInr*^74^ mutant elevates cysteine, taurine, and reduced-glutathione, which may favor somatic maintenance by managing ROS [49,63–65]. The Type II mutant at the Kinase Insert Domain is substantially different. The females are very fecund, and young adults have reduced somatic methionine activity and few products of the TSP. Yet, their mortality is remarkably low [4]. This result may arise through longevity assurance mechanisms associated with polyamine synthesis. As well, the methionine metabolism of these flies is flexible, where aged Type II adults increase their production of TSP. Remarkably, the different Type I and Type II metabolic syndromes arise from the placement of a single amino acid substitution within the insulin-like receptor. Mutation of the kinase insert domain, which produces insulin sensitive receptors, reduces somatic methionine cycle activity in young adults, while canonical, insulin resistant longevity alleles of *dInr* have excess methionine.

The ability to efficiently adapt metabolism by substrate utilization in response to nutrient intake and timing is a form of metabolic flexibility. Metabolic flexibility was first described in lean humans as they alter the fuels they burn when fasting when simultaneously they are infused with insulin, in contrast to the static fuel use seen in obese individuals [66,67]. Metabolic flexibility may explain why mild insulin resistance, which is seen in a variety of long-lived mice, is not necessarily an indicator of poor health [68]. While the concept of metabolic flexibility is typically applied on the scale of daily physiology as tissues transit between carbohydrate and lipid metabolism, here we envision metabolic flexibility on the scale of aging. In the Type II KID mutant, this flexibility permits adults to adjust methionine metabolism from a MetR-like state when young to a state where methionine feeds the transsulfuration pathway as they age.

## Materials and methods

### Fly stocks and culture

The insulin receptor mutants were generated in Yamamoto *et al* [4]: *dInr*^+[HR]^ (wildtype, accession ‘29B’), *dInr*^353[HR]^, *dInr*^E19[HR]^, *dInr*^74[HR]^. All alleles were generated by ends-out gene replacement. Wildtype accession ‘29B’ is a gene replacement of the wildtype allele back into its originating stock (*w*Dahomey, *w*Dah, source: L. Partridge UCL, UK). Mutant alleles were produced by nucleotide substitutions of the *w*Dah wildtype allele to alter the targeted amino acid. All replacemtent *dInr* alleles were maintained as third chromosome (TM6) balanced stocks. Mutant genotypes for all analyses were generated by crossing *dInr*^353^/TM6B *Tb,Sb,e* to *dInr*^wt^, recovering F1 *dInr*^353^/*dInr*^wt^, and by crossing *dInr*^E19^/*Tb,Sb,e* to *dInr*^74^/*Tb,Sb,e*, recovering trans-heterozygote *dInr*^E19^/*dInr*^74^. Other stocks used in the epistasis analysis: *gnmt*-RNAi: w[1118]; P{GD10563}v25983 (VDRC); *samtor*-RNAi: P{KK107414}VIE-260B (VDRC); Ahcy13-RNAi: *y*^1^ sc* *v*^1^ *sev*^21^; P{TRiP.HMS05799}attP40 (BDSC #67848); S6K: w[1118]; P{w[+mC]=UAS-S6k.TE}2 (BDSC #6912). tubulinGal4; tubulinGal80ts stock is a gift from the lab of Dr. Norbert Perrimon.. Each of the VDRC and BDSC lines were backcrossed into *w*Dah; + / + ; + /+ for five generations. Flies were reared at 25°C, 40% RH, 12L:12D on standard food media: cornmeal (5.2%), sugar (11.0%), autolyzed yeast (2.5%; SAF brand, Lesaffre Yeast Corp., Milwaukee, WI, USA.), agar (0.79%) (w/v in 100 mL water) with 0.2% Tegosept (methyl4-hydroxybenzoate, Sigma, St Louis, MO, USA), with baker’s yeast supplemented to the food surface.

### Infection survival assay

We followed the protocols of Troha and Buchon [69] to orally infect adults with *Pectinobacterium carotovora carotovora* (strain ECC15). ECC15 was grown in shaking culture at 29C overnight, pelleted by centrifugation (10 min at 2400g, 4C) and resuspended in sterile PBS to OD600. Treatment conditions were initiated with 8-to-9-day-old adult females, collected over a period of 48 hours post-eclosion in the presence of males. The beginning of the treatment period is designated as survival age zero. Cohorts of 100 adults for each genotype were evenly distributed across 10 food vials per treatment group (PBS control, ECC15). A volume of 100 μL of either sterile PBS or Ecc15 suspension was added to a round filter paper placed on top of the food within each vial. Prior to the treatment, flies were starved for 2 hours in empty vials, then placed in ECC15 or control treated vials and retained for 18–24 hours. Subsequently, adults were flipped into fresh, uninfected food daily, at which time dead flies were removed and counted. Kaplan-Meier survival curves are plotted with right-censored data at survival age 47 days. Cox-proportional survival analysis evaluated genotype, treatment and genotype-by-treatment interaction.

### RNA-seq data and analysis

Flies eclosed and mated over a 48-hour period and females of each genotype (wildtype, *dInr*^E19^/*dInr*^74^, *dInr*^353^/*dInr*^wt^) were then collected into six biological replicates of 20 females. Each replicate was lysed in Trizol using a bead Tissuelyzer (Qiagen) at room temperature. RNA was extracted from each sample by NH_4_OAc and EtOH precipitation. RNA concentration (ng/μl) and purity (absorbance: 260nm/280nm) was confirmed with a Nanodrop spectrophotometer (ThermoFisher Scientific). Samples were sent to Genewiz for library preparation (Illumina, RNA with PolyA selection) and Illumina HiSeq 2x150 bp sequencing. Returned reads were processed on the Basepair platform for RNA-Seq QC, alignment, and counts. Differential expression, gene set enrichment, K-means enrichment and transcription factor binding site enrichment were generated with iDEP (http://bioinformatics.sdstate.edu/idep96/).

### Steady state metabolomics

Tissue for analysis of steady state metabolomic profiles were collected from females at age 15d and 30d. Females eclosed in the presence of males and were flipped into female only vials from age 2 days until sample collection. Six biological replicates of each genotype (*dInr*^wt^/*dInr*^wt^, *dInr*^E19^/*dInr*^74^, *dInr*^353^/*dInr*^wt^) were generated with 10 females per sample. Females aged to 15 and 30d were collected between 9 and 10 AM, anesthetized with CO_2_ and dissected to remove the abdomen to avoid signal from eggs. The somatic tissue was flash frozen in liquid nitrogen and stored at -80. Samples were shipped on dry ice to the Northwestern Metabolomics Research Center (NWMRC) at the University of Washington, Seattle. Protein from each biological sample was extracted at the NWMRC and quantified by Targeted LC-MS across a 360-feature set (https://northwestmetabolomicsorg.wpcomstaging.com/300-aqueous-metabolites/). The analysis returns Quality Control statistics and relative concentration of each detected metabolite based on isotope-labeled internal standards. Data processing and analyses were performed using metaboanalyst (http://www.metaboanalyst.ca/MetaboAnalyst/). Metabolites that were not detected in 40% of the samples were excluded from the analysis. Data were normalized to the median (per sample) and processed through log transformation. Heat map and hierarchical clustering was generated using Pearson correlations and Ward’s method. Metabolite Set Enrichment Analysis (MSEA) was performed using Metaboanalyst which uses the KEGG (http://www.genome.jp/kegg/pathway.html) pathway database. Metabolite sets containing at least five compounds were employed in the analysis. MSEA uses *globaltest* to test associations between metabolite sets and the outcome [70]. The algorithm uses a generalized linear model to compute a ‘Q-stat’ for each metabolite set. The Q-stat is calculated as the average of the Q values calculated for the each single metabolites; while the Q value is the squared covariance between the metabolite and the outcome [70].

### Labeled metabolite profile

Tissue for analysis of methionine labeling was collected from females at 10 days of age. Females eclosed in the presence of males. Flies were transferred to vials with fresh food for two days, after which females alone were separated into vials with chemically defined food (CDF) containing 1 mM unlabeled methionine for two days. At day five, females were transferred to CDF with 1 mM ^13^C5-methionine for 5 days and flipped every 2 days onto fresh CDF containing labeled methionine, or in parallel, maintained on CDF with unlabeled methionine (to correct for the natural abundance of ^13^C isotopes).

On day ten, we generated six biological replicates of each genotype (*dInr*^wt^/*dInr*^wt^, *dInr*^E19^/*dInr*^74^, *dInr*^353^/*dInr*^wt^), with 15 females per sample. Between 9 and 10 AM, the flies were transferred to empty vials containing a piece of Kimwipe soaked with 800 µL of 5% sucrose solution and kept for four hours (to clean their intestines of the labeled food). After four hours, whole (non-dissected) flies were anesthetized with CO₂, placed into pre-weighed vials, quickly weighed, flash-frozen in liquid nitrogen, and stored at −80 °C.

Intracellular metabolites were extracted using 1.2 mL of cold (−80 °C) 80% (v/v) aqueous methanol. Flies were homogenized using 0.5 mm zirconium beads (via three consecutive rounds of homogenization in a Bullet Blender in the cold room), and the insoluble material in lysates was centrifuged at 10,000 rpm for 5 minutes. The resulting supernatant was evaporated using a SpeedVac.

For metabolomic analysis, samples were mixed with −20 °C 80%:20% methanol:water (extraction solvent), vortexed, and immediately centrifuged at 16,000 x g for 20 min at 4°C. The supernatant was collected for LC-MS analysis. LC was performed on an Xbridge BEH amide HILIC column (Waters) with a Vanquish UHPLC system (Thermo Fisher). Solvent A was 95:5 water: acetonitrile with 20 mM ammonium acetate and 20 mM ammonium hydroxide at pH 9.4. Solvent B was acetonitrile. The gradient used for metabolite separation was 0 min, 90% B; 2 min, 90% B; 3 min, 75%; 7 min, 75% B; 8 min, 70% B, 9 min, 70% B; 10 min, 50% B; 12 min, 50% B; 13 min, 25% B; 14 min, 25% B; 16 min, 0% B, 21 min, 0% B; 21 min, 90% B; and 25 min, 90% B. MS analysis was performed on a Orbitrap Exploris 480 mass spectrometer (Thermo Fisher) by electrospray ionization with the parameters as: scan mode, full MS; spray voltage, 3.6 kV (positive) and −3.2 kV (negative); capillary temperature, 320 °C; sheath gas, 40 arb; aux gas, 7 arb; resolution, 120,000 (full MS); scan *m*/*z* range, 70–1,000.

Raw LC–MS data files were converted to mzXML format files by ProteoWizard and analyzed using El-MAVEN Software (Elucidata; elucidata.io). Isotope labeling was corrected for the natural abundance of ^13^C isotopes.

### Demographic epistasis analysis

Females for survival analysis were collected as F1 progeny over a 48-hour emergence period in the presence of males, then separated into all female demography cages with 125 adults in each of three cages per genotype. Demography cages were made from 1 L clear food service containers with a ventilated lid, a gasket-covered aperture to provide access to remove dead flies and a port near the cage bottom that opened to a plastic tube affixed a standard glass media vial with 3 ml of standard Drosophila diet. Every two days, dead flies were removed by aspiration and counted, and fresh food vials were provided.

Genotypes for survival analyses were produced by replacing the balancer third chromosome of *dInr*^353^ with the *tubulin*-Gal4.Gal80 third chromosome, followed by replacing the second chromosome of offspring with the targeted UAS-RNAi or over-expression. Females from these crosses were set up in controlled density bottles to lay eggs. Hatching larvae were reared at 25°C to suppress transgene expression. Emerging adults were shifted to the permissive temperature (29°C) and sorted into demography cages, maintained at 29°C. Wildtype F1 control and *dInr*^353^ single mutant genotypes were generated by replacing the third chromosome balancer with the *tubulin*-Gal4,Gal80 third chromosome. Cox proportional hazard analysis evaluated the impact of each genotype and their interaction.

## Supporting information

S1 Table Differentially expressed genes. (XLSX)

S2 Table Gene set enrichment, genotypes combined. (XLSX)

S3 Table Altered metabolites on day 15 and day 30. (XLSX)

S1 Fig Methionine cycle metabolites of *dInr*^E19/74^, *dInr*^353/+^ and *dInr*^+/+^. (TIF)
