# Supplementary figures and images for: An insulin-sensitive Drosophila insulin-like receptor mutant remodels methionine metabolism to extend lifespan
Source: PLoS Genet. 2025 Jun 16;21(6):e1011640. doi: 10.1371/journal.pgen.1011640 (PMC12208457; doi:10.1371/journal.pgen.1011640)

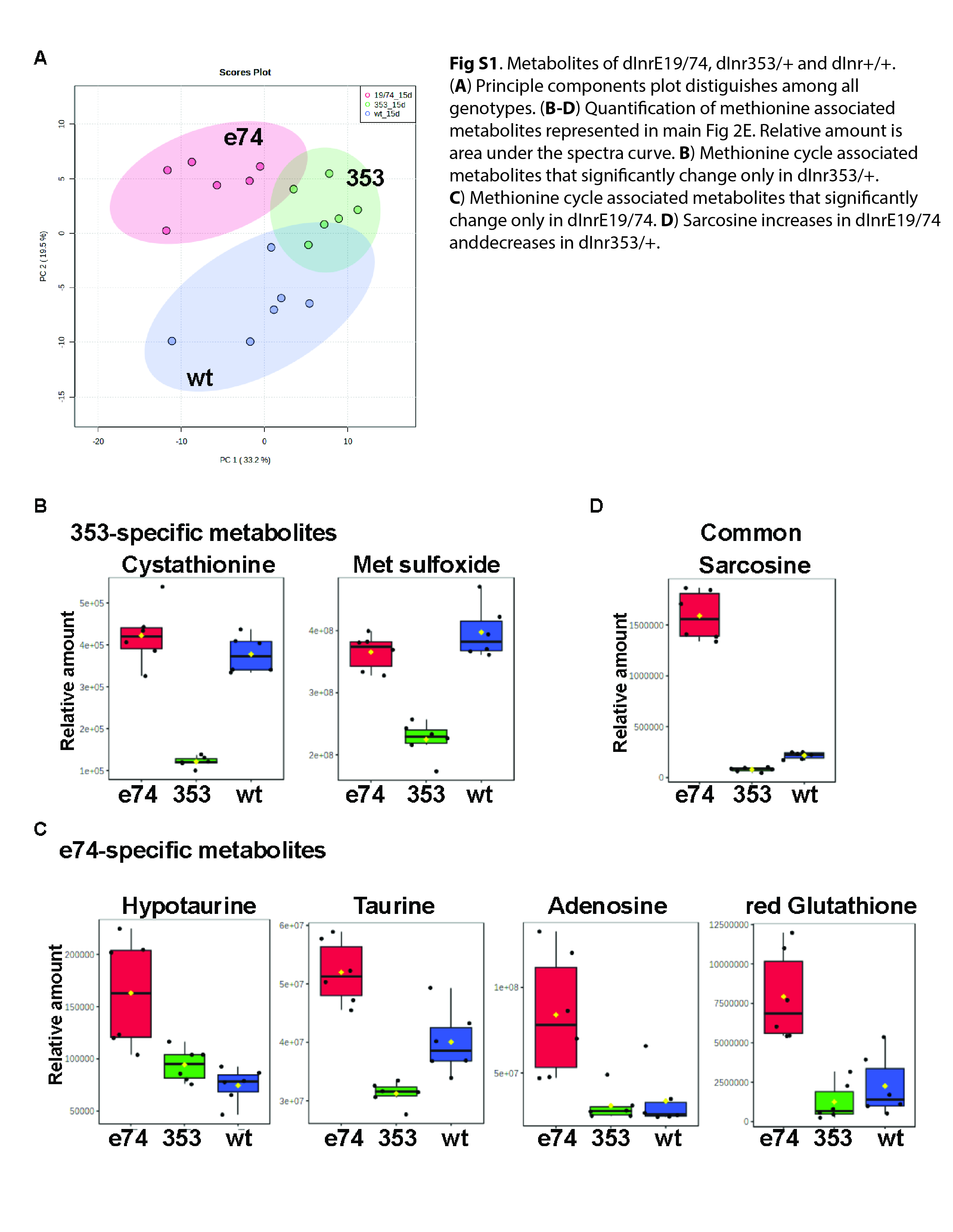

Supplement: S1 Fig — (TIF) [file pgen.1011640.s004.tif]
